# A Fiber-Rich Diet and Radiation-Induced Injury in the Murine Intestinal Mucosa

**DOI:** 10.3390/ijms23010439

**Published:** 2021-12-31

**Authors:** Dilip Kumar Malipatlolla, Sravani Devarakonda, Piyush Patel, Fei Sjöberg, Ana Rascón, Rita Grandér, Viktor Skokic, Marie Kalm, Jolie Danial, Eva Mehdin, Malin Warholm, Henrietta Norling, Andrea Stringer, Malin E. V. Johansson, Margareta Nyman, Gunnar Steineck, Cecilia Bull

**Affiliations:** 1 The Division of Clinical Cancer Epidemiology, Department of Oncology at the Institute of Clinical Sciences, Sahlgrenska Academy at the University of Gothenburg, 413 90 Gothenburg, Sweden; dilip.kumar.malipatlolla@gu.se (D.K.M.); sravani.devarakonda@gu.se (S.D.); piyush.patel@gu.se (P.P.); fei.sjoberg@microbio.gu.se (F.S.); rita.grander@neuro.gu.se (R.G.); viktor.skokic@gu.se (V.S.); joliiie.d@gmail.com (J.D.); gusmehev@student.gu.se (E.M.); malinwarholm@hotmail.com (M.W.); hettan93@gmail.com (H.N.); gunnar.steineck@oncology.gu.se (G.S.); 2 Department of Infectious Diseases at the Institute of Biomedicine, Sahlgrenska Academy at the University of Gothenburg, 413 90 Gothenburg, Sweden; 3 Department of Food Technology, Engineering and Nutrition, Lund University, 221 00 Lund, Sweden; ana.rascon@glucanova.com (A.R.); margareta.nyman@food.lth.se (M.N.); 4 Department of Pharmacology at the Institute of Neuroscience and Physiology, Sahlgrenska Academy at the University of Gothenburg, 413 90 Gothenburg, Sweden; marie.kalm@astrazeneca.com; 5 School of Pharmacy and Medical Sciences, University of South Australia, Adelaide, SA 5000, Australia; andrea.stringer@unisa.edu.au; 6 Department of Medical Biochemistry and Cell Biology at the Institute of Biomedicine, Sahlgrenska Academy at the University of Gothenburg, 413 90 Gothenburg, Sweden; malin.johansson@medkem.gu.se

**Keywords:** dietary fiber, oat, irradiation, colon, intestine, bacteria, cytokines

## Abstract

Dietary fiber is considered a strong intestinal protector, but we do not know whether dietary fiber protects against the long-lasting mucosal damage caused by ionizing radiation. To evaluate whether a fiber-rich diet can ameliorate the long-lasting pathophysiological hallmarks of the irradiated mucosa, C57BL/6J mice on a fiber-rich bioprocessed oat bran diet or a fiber-free diet received 32 Gray in four fractions to the distal colorectum using a linear accelerator and continued on the diets for one, six or 18 weeks. We quantified degenerating crypts, crypt fission, cell proliferation, crypt survival, macrophage density and bacterial infiltration. Crypt loss through crypt degeneration only occurred in the irradiated mice. Initially, it was most frequent in the fiber-deprived group but declined to levels similar to the fiber-consuming group by 18 weeks. The fiber-consuming group had a fast response to irradiation, with crypt fission for growth or healing peaking already at one week post-irradiation, while crypt fission in the fiber-deprived group peaked at six weeks. A fiber-rich diet allowed for a more intense crypt cell proliferation, but the recovery of crypts was eventually lost by 18 weeks. Bacterial infiltration was a late phenomenon, evident in the fiber-deprived animals and intensified manyfold after irradiation. Bacterial infiltration also coincided with a specific pro-inflammatory serum cytokine profile. In contrast, mice on a fiber-rich diet were completely protected from irradiation-induced bacterial infiltration and exhibited a similar serum cytokine profile as sham-irradiated mice on a fiber-rich diet. Our findings provide ample evidence that dietary fiber consumption modifies the onset, timing and intensity of radiation-induced pathophysiological processes in the intestinal mucosa. However, we need more knowledge, not least from clinical studies, before this finding can be introduced to a new and refined clinical practice.

## 1. Introduction

Despite the significant improvements of radiotherapy in cancer treatment, radiation-induced injury to the normal, non-tumorigenic tissue is still a dose- and treatment-limiting issue. The growing number of cancer survivors who are alive decades after their cancer treatments have further exposed the severity of the problem. Symptoms from normal-tissue damage may arise both acutely and long after the completion of radiotherapy. If the symptoms arise as a consequence of pelvic radiotherapy, they are recognized as manifestations of pelvic radiation disease [1]. It is estimated that millions of cancer survivors worldwide are suffering from chronic intestinal dysfunction as a result of their cancer treatment, with an incidence that exceeds that of Crohn’s disease [1]. We have previously identified 28 late gastrointestinal symptoms after pelvic radiotherapy and categorized them into five main syndromes: excessive mucus discharge, excessive gas discharge, fecal leakage syndrome, fecal urgency syndrome and blood discharge [2]. The underlying mechanisms of these syndromes have not been elucidated, but radiation-induced DNA damage, both directly and through the production of reactive oxygen species, plays a vital role in the development of the normal-tissue injury after pelvic radiotherapy [3]. Proliferating stem cells are vulnerable to ionizing radiation and will undergo apoptosis within hours following exposure [4]. It is believed that the elimination of stem cells causes crypt loss and disrupts the integrity of the epithelial monolayer, resulting in increased permeability of the gut wall. Radiation also appears to disrupt the tight junctions between cells, additionally increasing permeability. Furthermore, radiation-induced damage to capillaries in the intestinal wall may lead to ischemia. While the acute effects of ionizing radiation on the intestinal mucosa have been studied in detail, including the activation of the mucosal immune system (reviewed in [5]), much less is known about the long-term effects. This is in part due to the current limitations of existing animal models, and few studies have been performed on biopsies from long-term cancer survivors.

The use of dietary fiber to improve gastrointestinal health is gaining ground [6,7,8]. A diet rich in fiber helps the colon to nurture beneficial bacteria and stimulate stem cells to proliferate and maintain epithelial homeostasis [9,10]. The colonic fermentation of dietary fiber, including β-glucans, form short-chain fatty acids such as butyrate that are claimed to be important mediators of the beneficial effects of dietary fiber on intestinal health [11]. Experiments using fiber-free diets suggest that a lack of dietary fiber disrupts cell proliferation, crypt fission and crypt morphology [12,13]. In light of this, it is concerning that a common piece of advice to cancer survivors with gastrointestinal problems is to reduce fibers [14]. The advice is probably sprung from the knowledge that consuming a large amount of fiber might produce excessive gas and abdominal pain. However, in the long run, the advice could be counterproductive and even harmful. In this study, we sought to determine whether the enrichment of dietary fiber in the diet, or a lack thereof, would affect the long-term outcome concerning several gross parameters of radiation-induced mucosal injury and repair, such as crypt degeneration, crypt fission and poor mucosal barrier integrity reflected by bacterial infiltration. Modeling pelvic radiotherapy in mice, we delivered radiation to the mouse colorectum in multiple fractions with a clinically relevant dose rate using the clinic’s linear accelerators. This approach allows for the long-term follow-up of pathophysiological processes and for the reparation of mechanisms after irradiation. We focused on dietary supplementation with fiber-rich bioprocessed oat bran containing high levels of β-glucans that are readily fermented into short-chain fatty acids in the colon. 

## 2. Results

### 2.1. Animal Health Status and Body Weight

**Figure 1:**
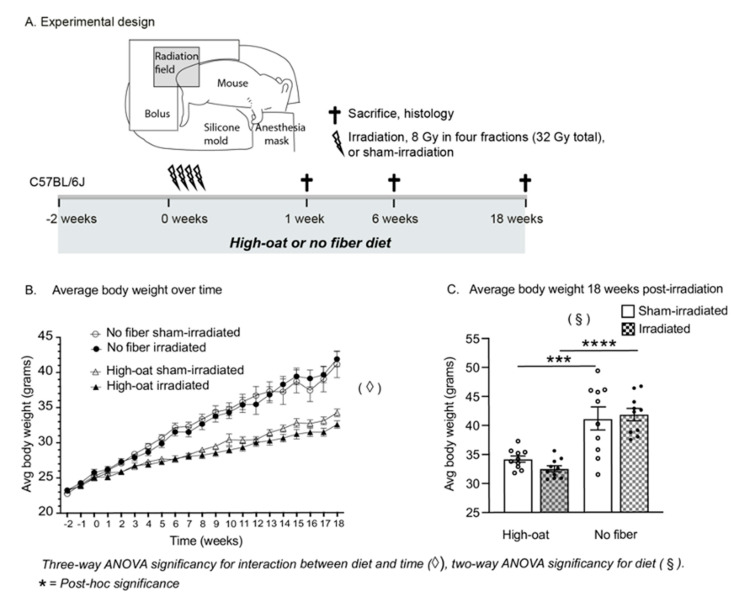
(**A**). Experimental design: 9 week old male C57BL/6J mice were fed either a fiber-rich bioprocessed oat bran diet (“High-oat”) or a fiber-deprived diet (“No fiber”) for two weeks before irradiation or sham-irradiation. During irradiation, 32 Gray was given to the colorectum with a 6 MV linear accelerator, in four fractions of 8 Gy spaced by 12 h. The diets were continued throughout the experiment, and mice were euthanized 1 week (acute time point), 6 weeks (intermediate time point) or 18 weeks (late time point) after irradiation. The colorectal tissues were harvested for the collection of various metrics on injury and repair. (**B**). Weight curves of mice during the 20 weeks of the dietary intervention and up to 18 weeks after irradiation. Fiber-deprived mice gained more weight over time than mice fed with bioprocessed oat bran (*p* < 0.0001 for interaction between time and diet). (**C**). At 18 weeks after irradiation, mice on a fiber-free diet had gained significantly more weight than mice on a fiber-rich diet, and irradiation did not affect their weight gain. *** *p* < 0.0006, **** *p* < 0.0001. Data are shown as mean ± S.E.M.

The experimental design is shown in Figure 1A. All of the mice were inspected and weighed every week (Figure 1B). A three-way mixed ANOVA, with time as a within-subjects factor, diet and irradiation as between-subjects’ factors and a Geisser-Greenhouse correction for the lack of sphericity, was conducted. A significant main effect of time was noted (*p* < 0.0001) as well as a significant time x diet interaction (*p* < 0.0001), with body weight increasing for all groups over the 18-week time period but significantly more so for the two no-fiber diet groups compared to the two High-oat diet groups. The increase in body weight with the No-fiber diet compared to the High-oat diet was also confirmed by a significant main effect of diet (*p* < 0.0001). No other effects or interactions were found. At 18 weeks post-irradiation a two-way ANOVA confirmed the strong effect of diet on body weight (Figure 1C; *p* < 0.0001), with an increase in body weight in the No-fiber irradiated mice compared to the High-oat irradiated mice (*p* < 0.0001, Bonferroni post-hoc) and in the No-fiber sham-irradiated mice compared to the High-oat sham-irradiated mice (*p* = 0.0006, Bonferroni post-hoc). 

### 2.2. Pronounced Early Crypt Degeneration in Irradiated No-Fiber Animals

**Figure 2:**
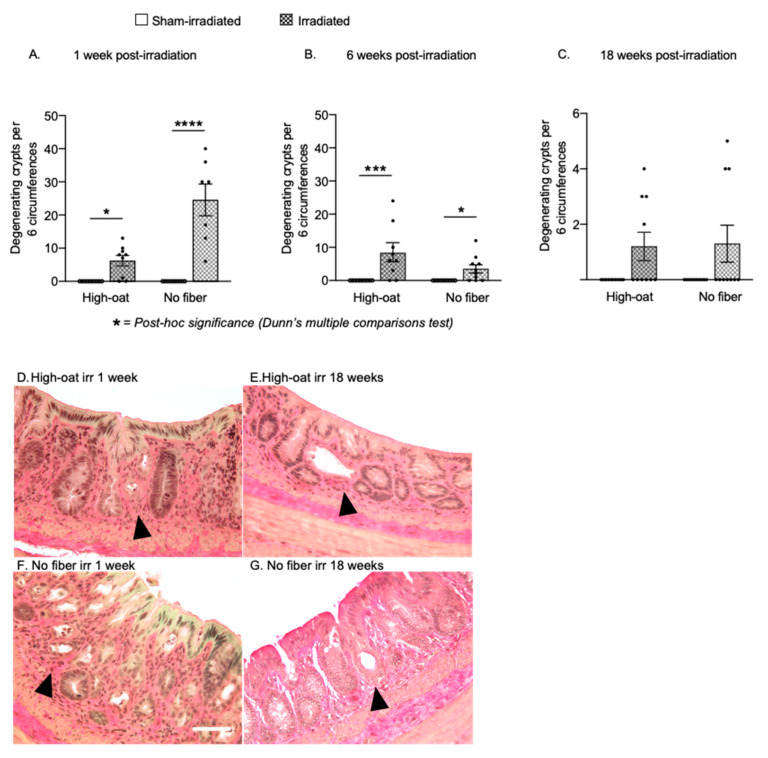
Number of degenerating crypts at 1 week, 6 weeks and 18 weeks after irradiation. Degenerating crypts were never seen in sham-irradiated animals. (**A**). More crypt degeneration was seen in the fiber-deprived animals (“No fiber”) than the oat-fed animals (“High-oat”) at one week after irradiation. * *p* = 0.0191 and **** *p* < 0.0001. (**B**). Although the initial high rate of crypt degeneration in the irradiated No-fiber mice had declined by 6 weeks post-irradiation, crypt degeneration still occurred in both diet groups. *** *p* = 0.0008 and * *p* = 0.0205. (**C**). At 18 weeks, the High-oat and No-fiber irradiated mice both had similar and low numbers of degenerating crypts. (**D**–**G**). Micrographs (40× magnification) of colorectal mucosa stained with Verhoeff’s Elastic Stain. Arrowheads depict examples of degenerating crypts. Scale bar 50 μm. Data are shown as mean ± S.E.M.

A total of six sections per animal were analyzed for degenerating crypts at 1, 6 and 18 weeks post-irradiation (Figure 2). In the literature, the term “crypt abscess” is sometimes encountered when describing degenerating crypts. We refrain from using the term here since it refers to the accumulation of inflammatory cells inside the crypt, not the withering of the crypt itself. We did not observe any crypt degeneration in sham-irradiated mice at any time point. At one week after irradiation (Figure 2A), the High-oat mice had several crypt degenerations per six circumferences (High-oat irradiated versus sham-irradiated *p* = 0.0191, Dunn’s post-hoc), an effect that was even more pronounced in the No-fiber irradiated mice (No-fiber irradiated versus sham-irradiated, *p* < 0.0001, Dunn’s post-hoc). At 6 weeks post-irradiation, the number of degenerating crypts had declined considerably in the No-fiber irradiated animals, while the number of degenerating crypts in the High-oat animals remained approximately the same (No-fiber irradiated versus sham-irradiated, *p* = 0.0205 and High-oat irradiated versus sham-irradiated, *p* = 0.0008, Dunn’s post-hoc). At 18 weeks, there was still an effect of irradiation on crypt degeneration (*p* = 0.0408, Kruskal-Wallis), but at that point the majority of irradiated animals had zero counts in the six circumferences analyzed per mouse (High-oat irradiated versus sham-irradiated, *p* = 0.1209 and No-fiber irradiated versus sham-irradiated, *p* = 0.2414, Dunn’s post-hoc). As noted previously [15], the condition of the irradiated mucosa varied within the same animal, with areas with an almost normal physiology flanking areas with obvious pathophysiology. For unknown reasons, some animals within the same dietary group exhibited worse pathophysiology than others, similar to the variation between irradiated human subjects. 

### 2.3. Increased Crypt Fission after Pelvic Irradiation

**Figure 3:**
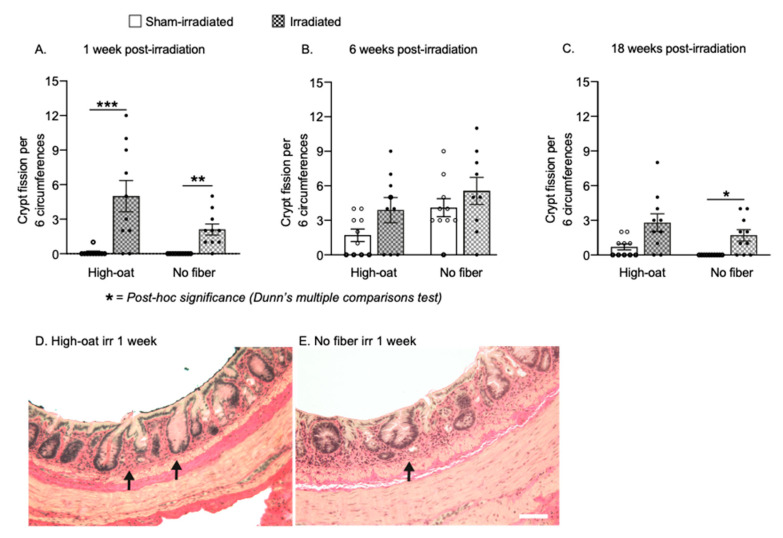
Number of crypt fissions per six circumferences at 1 week, 6 weeks and 18 weeks after irradiation. (**A**). An increase in the number of crypt fissions was observed in both the diet groups one week after irradiation, with a more pronounced increase in the High-oat group. *** *p* = 0.001 and ** *p* = 0.0034. (**B**). At six weeks, there was slight, but not statistically significant increase in the number of crypt fissions in both irradiated diet groups, and more crypt fissions in the animals fed the No-fiber diet. Both control groups had a higher baseline than the control groups at 1 and 18 weeks. (**C**). At 18 weeks, there were still more crypt fissions in the irradiated mice of both diet groups compared to the sham-irradiated mice. * *p* = 0.0157. (**D**,**E**). Micrographs (40× magnification) of colorectal mucosa stained with Verhoeff’s Elastic Stain. Arrows depict examples of crypt fissions. Data are shown as mean ± S.E.M.

At 1-week post-irradiation, there was a substantial increase in crypt fission events in the irradiated High-oat mice compared to sham-irradiated mice (Figure 3A; *p* = 0.001, Dunn’s post-hoc). There was also an increase in crypt fission events in the No-fiber irradiated mice, while no crypt fission events were observed in the six circumferences analyzed per sham-irradiated mouse (Figure 3A; *p* = 0.0034, Dunn’s post-hoc). At six weeks post-irradiation, a *p*-value of 0.0545 with the Kruskal-Wallis test and a visual inspection of the data suggested a slight radiation-induced increase in both groups but also a higher baseline (Figure 3B). At 18 weeks, both irradiated groups still had more crypt fissions than their sham-irradiated controls, although only the No-fiber irradiated group reached statistical significance (Figure 3C; No-fiber irradiated versus sham-irradiated, *p* = 0.0157).

### 2.4. A Fiber-Rich Diet Promotes a Lasting Cell Proliferation Response in the Crypts

**Figure 4:**
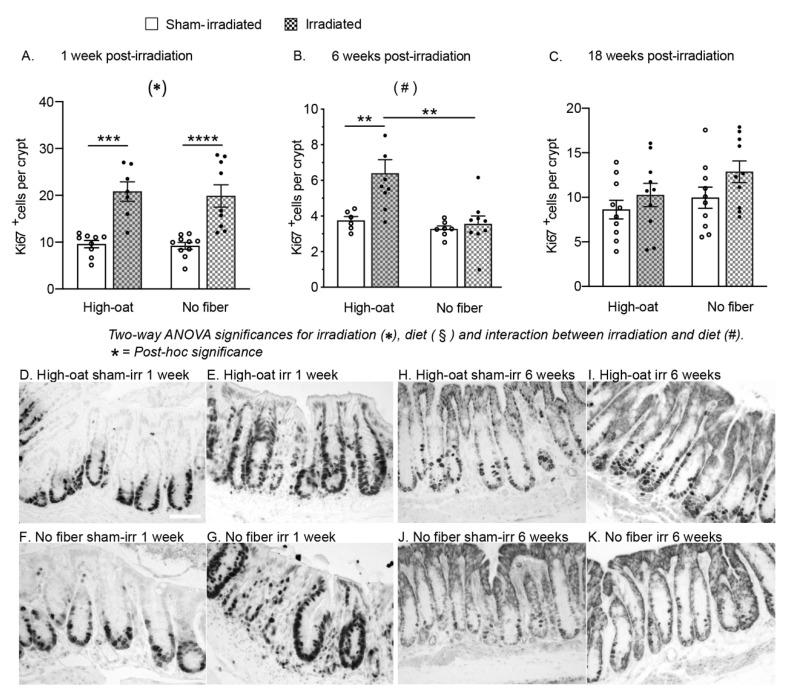
The average number of Ki67-positive proliferating cells per mucosal crypt in mice fed a fiber-rich or a fiber-free diet. (**A**). An irradiation-induced increase in the number of Ki67-positive cells was found in both the High-oat and No-fiber animals at one week after irradiation. *** *p* = 0.0002, **** *p* = 0.0001. (**B**). At six weeks, an increase in the Ki67-positive cells was still observed in the High-oat irradiated mice but not in the No-fiber mice. ** *p* = 0.0088, ** *p* = 0.0015. (**C**). At 18 weeks, there was a slight increase in crypt cell proliferation in both irradiated groups, although this was not statistically significant. (**D**–**K**). Micrographs (40× magnification) of proliferating cells visualized with immunohistochemistry using an antibody against Ki67 and a DAB stain. Scale bar 50 μm. Data are shown as mean ± S.E.M.

An irradiation-induced increase in the number of proliferating Ki67^+^ cells in the crypts was observed in both of the diet groups at 1 week (Figure 4A; *p* < 0.0001 for irradiation, two-way ANOVA). The cell proliferation was increased in both irradiated groups (High-oat irradiated mice versus sham-irradiated, *p* = 0.0002, and No-fiber irradiated versus sham-irradiated, *p* = 0.0001, Bonferroni post-hoc). At 6 weeks, the effect of irradiation on proliferation was dependent on diet (Figure 4B; *p* = 0.0117 for interaction diet x irradiation, two-way ANOVA), with a notable increase in cell proliferation only in the High-oat irradiated group (High-oat irradiated mice versus sham-irradiated, *p* = 0.0088, and High-oat irradiated versus No-fiber irradiated, *p* = 0.0015, Bonferroni post-hoc). At 18 weeks, irradiation had a small effect on cell proliferation levels that did not reach the criteria for statistical significance (Figure 4C; *p* < 0.0641 for irradiation, two-way ANOVA).

### 2.5. Dietary Fiber Does Not Protect against a Long-Lasting Reduction in Crypts after Irradiation

**Figure 5:**
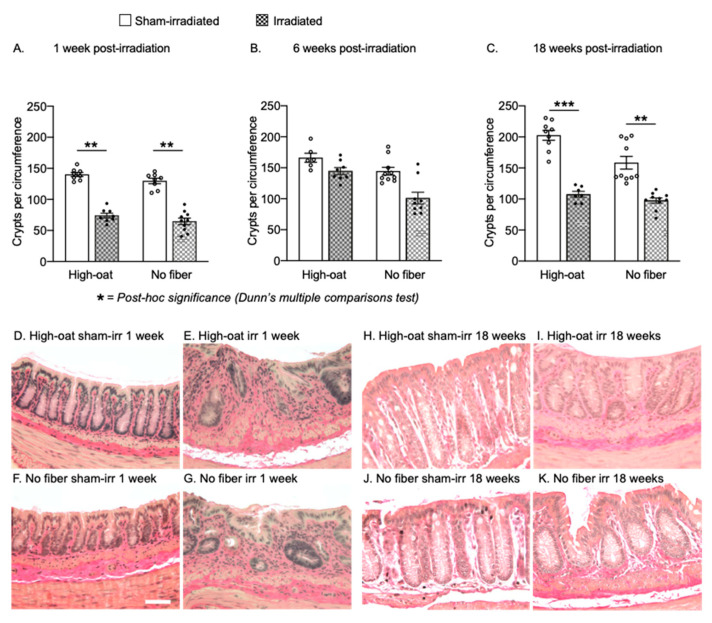
Crypts per circumference over time after irradiation. (**A**). A decrease in the number of crypts per circumference was seen in the High-oat and the No-fiber mice compared to the sham-irradiated mice at one week. ** *p* = 0.0025 and ** *p* = 0.0017. (**B**). At six weeks, the High-oat irradiated animals appeared to have more crypts compared to the No-fiber irradiated mice (*p* = 0.0771). (**C**). At 18 weeks, both the High-oat and the No-fiber irradiated animals had similar numbers of crypts. *** *p* = 0.0008 and ** *p* = 0.0025. (**D**–**K**). Representative micrographs (40× magnification, Verhoeff’s Elastic Stain) of crypts in the colorectal mucosa. Data are shown as mean ± S.E.M.

At 1 week post-irradiation, there was a loss of crypt in both the High-oat irradiated mice (Figure 5A; *p* = 0.0025, Dunn’s post-hoc) and No-fiber irradiated mice (Figure 5A; *p* < 0.0017, Dunn’s post-hoc) compared to their sham-irradiated controls. At 6 weeks post-irradiation, the loss of crypts was still significant (Figure 5B; *p* = 0.0022, Kruskal-Wallis) but not as obvious, especially in the High-oat diet group, and Dunn’s post-hoc analysis returned a value of *p* = 0.0771 for High-oat irradiated versus No-fiber irradiated. At the 18-weeks timepoint, considerably fewer crypts were observed again in the irradiated animals (Figure 5C; high-oat irradiated versus sham-irradiated, *p* = 0.0008, and No-fiber irradiated versus sham-irradiated, *p* = 0.0025, Dunn’s post-hoc), with a larger drop from a higher baseline in the High-oat irradiated animals. Notably, at this timepoint, both irradiated groups had similar numbers of crypts per circumference (Figure 5C; High-oat irradiated versus No-fiber irradiated, *p* > 0.9999, Dunn’s post-hoc). 

### 2.6. A Fiber-Rich Diet Completely Protects from Late Irradiation-Induced Bacterial Infiltration

**Figure 6:**
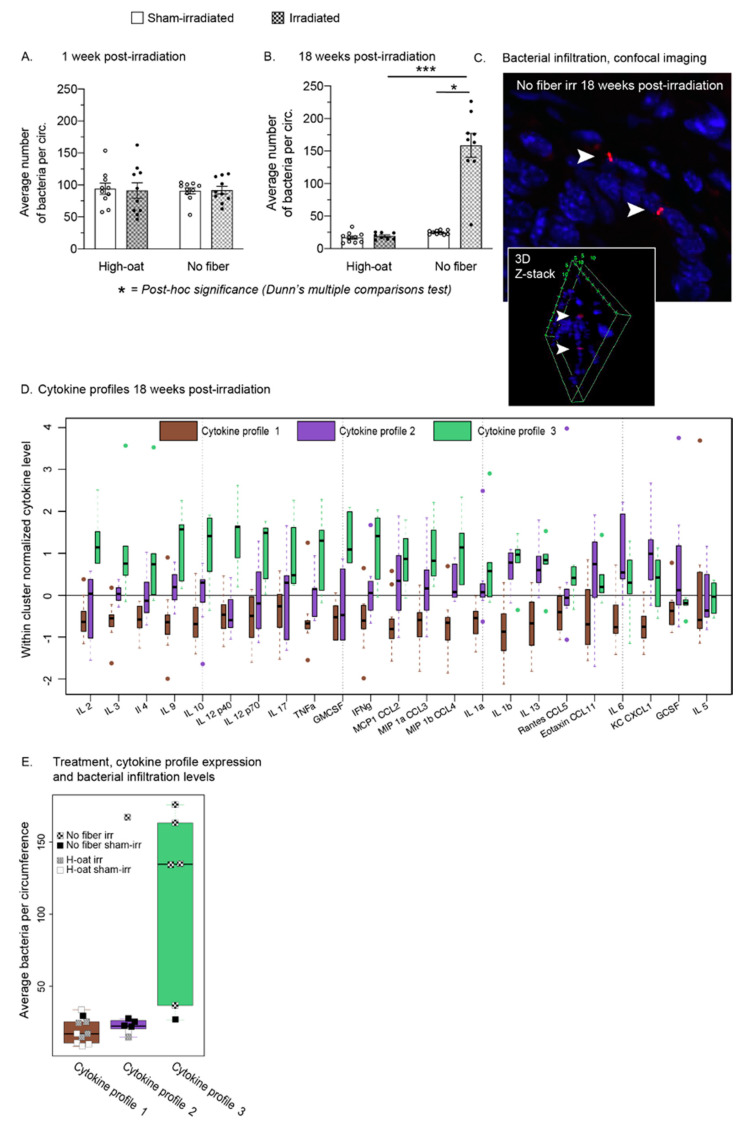
The average number of bacteria per circumference at 1 week and 18 weeks after irradiation. (**A**). Quantification of bacteria in the mucosa one week after irradiation confirmed that bacterial infiltration did not occur early after irradiation, and the absence of dietary fiber in the chow did not yet facilitate infiltration. (**B**). At 18 weeks after irradiation, a manyfold increase of bacterial presence in the mucosa was found in irradiated mice on a fiber-free diet. Irradiated mice consuming fiber-rich oat bran were entirely protected against irradiation-induced bacterial infiltration. * *p* = 0.0451 and *** *p* = 0.0005. (**C**). Confocal imaging of bacteria (arrowhead; bacteria in red, cell nuclei in blue) in the irradiated colorectum of a mouse fed a fiber-free diet. Z-stack imaging shows the location of bacteria within the tissue. Data are shown as mean ± S.E.M. (**D**). Boxplots of within-cluster normalized serum cytokine levels at 18 weeks post-irradiation. The mice expressed either one of three distinct cytokine profiles. (**E**). The majority of irradiated mice on a fiber-free diet, with higher levels of bacterial infiltration, were clustering into profile 3, with a more pro-inflammatory pattern than profile 1 and 2. In contrast, mice on a fiber-rich diet expressed profile 1, regardless of whether they had been irradiated or not. Boxplots with median and interquartile range.

Bacteria were quantified in the mucosa and submucosa at the acute time point (1 week post-irradiation) and the late time point (18 weeks post-irradiation). There were no differences in bacterial infiltration between the groups at 1 week post-irradiation (Figure 6A). At 18 weeks, there was a profound increase in bacterial infiltration in the No-fiber irradiated mice compared to their sham-irradiated controls (Figure 6B, *p* = 0.0451, Dunn’s post-hoc) and compared to the High-oat irradiated mice (Figure 6B; High-oat irradiated versus No-fiber irradiated, *p* = 0.0005, Dunn’s post-hoc). No increase in the High-oat irradiated mice was found. 

### 2.7. Three Distinct Serum Cytokine Profiles 18 Weeks after Irradiation

Serum cytokine levels of 23 cytokines were retrieved from an earlier study on the same mice, six mice per group [16]). Hierarchical clustering of the expression levels of the cytokines at 18 weeks post-irradiation resulted in three distinct patterns of cytokine expression levels (Figure 6D, cytokine profile 1–3, represented by green, purple and brown, respectively). A possible association between the three cytokine profiles produced and the four treatment groups was examined using Fisher’s exact test, resulting in a *p*-value < 0.001. High-oat sham-irradiated and high-oat irradiated mice expressed a similar cytokine profile (Figure 6E, cytokine profile 1), while No-fiber sham-irradiated and irradiated mice expressed two separate profiles. No-fiber sham-irradiated mice had a moderately pro-inflammatory profile (Figure 6E, cytokine profile 2), while No-fiber irradiated mice had a more pro-inflammatory profile (Figure 6E, cytokine profile 3). Moreover, there was an association between high levels of bacterial infiltration in the mucosa and cytokine profile 3.

### 2.8. Inflammatory Activity

**Figure 7:**
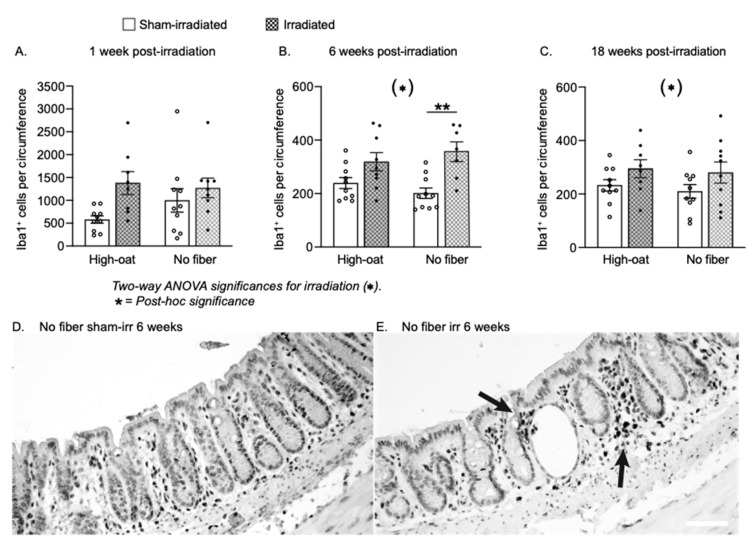
The average number of mucosal macrophages per circumference at 1 week, 6 weeks and 18 weeks after irradiation. (**A**). A potential increase in the number of macrophages was seen at one week in the High-oat irradiated mice compared to the High-oat sham-irradiated. (**B**). At 6 weeks, both diet groups had a greater abundancy of macrophages after irradiation than the sham-irradiated mice (*p* = 0.0002 for irradiation). ** *p* = 0.0022. (**C**). At 18 weeks, there were still more macrophages in the mucosa of irradiated mice of both diet groups (*p* = 0.0386 for irradiation). (**D**,**E**). Micrographs (20× magnification, scale bar 50 μm) of macrophages visualized with immunohistochemistry using an antibody against Iba1 and a DAB stain. Iba1^+^ macrophages were found in the mucosa of both sham-irradiated (**D**) and irradiated (**E**) animals but were more abundant in the irradiated animals and often clustered (arrows). Data are shown as mean ± S.E.M.

To further assess inflammatory activity, we quantified the number of Iba1-positive macrophages at 1, 6 and 18 weeks post-irradiation. A two-way ANOVA at 1 week post-irradiation returned *p*-values larger than 0.05 for all three factors (Figure 7A). At 6 and 18 weeks post-irradiation, the mucosa of irradiated mice contained more mucosal macrophages than the sham-irradiated, regardless of diet (Figure 7B,C; *p* = 0.0002 and *p* = 0.0386 respectively, two-way ANOVA), with the largest increase at 6 weeks in the No-fiber irradiated mice (Figure 5B; *p* = 0.0022, Bonferroni post-hoc). 

## 3. Discussion

The results from this study provide evidence that the pathophysiological processes triggered by ionizing irradiation in the distal bowels are modified by the diet. Importantly, our study demonstrated that irradiation caused bacterial infiltration into the mucosa with an onset long after the acute damage and that this could be completely prevented by a dietary approach. Moreover, the bacterial infiltration correlated with a specific pro-inflammatory cytokine profile. 

In the present study, the combination of a fiber-deprived diet and irradiation-induced damage resulted in a profound increase of bacteria penetrating the intestinal wall. That the bacterial penetration was not seen at the early time point but only occurred later on was unexpected, since bacterial penetration after irradiation has been suggested to be an acute phenomenon [17,18]. One explanation for this discrepancy might be the use of large single doses in many radiotherapy models instead of fractionated radiation. In the clinic, pelvic radiation is usually given in multiple, low-dose fractions to target the fast-dividing tumor cells while sparing the normal tissue. The sparing of tissue using fractionated radiation might mean that the dynamics of bacterial infiltration could be quite different in the clinical setting than what is expected from animal studies. This could have implications for cancer treatment and survival care and merits further investigation.

Fermentable dietary fiber promotes the thickness and integrity of the mucus layers lining the inside of the colonic tract, thereby protecting from inflammation caused by pathogens penetrating the intestinal wall [19]. If the microbiota is deprived of dietary fiber, the luminal environment becomes more favorable for gram-negative bacterial strains, and the substrate for bacterial energy-production shifts from dietary fiber to mucus glycoproteins. This results in the erosion of the mucus barrier, making the mucosa more susceptible to invading pathogens [10]. We have previously found that fiber deprivation increased the mucus-degrading activity in our mouse model, an effect that was exacerbated by irradiation (P. Patel et al., unpublished results). It is reasonable to assume that mucus erosion permitted the bacteria to penetrate the mucosa in the present study. However, mucus erosion has been suggested to be a fast process [19], while the penetration of bacteria was a late phenomenon in our mice and was not detectable one week after irradiation. An exposed epithelial layer may perhaps withstand digestion by host and bacterial enzymes for a limited period before the progression of pathophysiological processes eventually cause a barrier breach. Here, a barrier breach appeared to be considerably facilitated by irradiation; in our sham-irradiated animals, there was only a small influx of bacteria, while there was a manyfold increase in the fiber-deprived irradiated intestine. Irradiation-induced injury to the epithelial layer, such as loss of functional tight junctions, disruption of metabolic processes in the mucosa and/or other known and unknown pathological processes, may have reduced the intestinal resilience. In contrast, the integrity of the irradiated mucosa remained entirely protected from invading bacteria if the animals were fed a fiber-rich diet. Whether the diet also protected from other pathogens such as viruses and fungi, and whether a fiber-rich diet offers a similar protection against pathogens in irradiated cancer survivors, are important follow-up questions.

We were able to distinguish three distinct serum cytokine profiles expressed by the four treatments groups 18 weeks after irradiation. One profile had higher levels of nearly three-quarters of all cytokines measured when compared to the other two profiles. This cytokine profile was also the only one to display an increase of the IL-12 p40 subunit. The profile was expressed by the irradiated mice on a fiber-free diet and coincided with increased bacterial infiltration into the mucosa. Although the p40 subunit will form IL-12 when pairing with the p35 subunit, it can also pair with the p19 subunit instead and form IL-23. While both IL-12 and IL-23 are implicated in intestinal inflammation, IL-12 is believed to initiate inflammation following bacterial infiltration into the mucosa, while the IL-23 pathway plays a role in the development of chronicity [20]. Mice deficient in the p40 subunit are protected from colitis, and agents that interfere with p40 and the downstream pathways of both IL-12 and IL-23 are already in use, or are being evaluated as novel treatments, for the two major inflammatory bowel diseases (IBD), Crohn’s disease and Ulcerative Colitis [21]. IL-23 stimulates the production of IL-17-producing T cells, which maintain a pro-inflammatory environment. The pathogenicity of the Th17 cells is dependent on their production of IFN-γ, which increases the permeability of the intestinal barrier, and GM-CSF, which recruits granulocytes and monocytes [22,23]. These cytokines were also found to be elevated in the irradiated mice on a fiber-free diet. Our finding of a long-lasting pro-inflammatory serum cytokine profile, where p40 may play a role, suggests that lessons learned from the large bulk of IBD research can be useful when trying to understand the development and maintenance of chronic intestinal dysfunction after pelvic radiotherapy. However, to date, the presence of a similar, chronic low-grade inflammation with bacterial infiltration in the mucosa of irradiated pelvic cancer survivors has not been thoroughly investigated.

Crypt degeneration and crypt loss are hallmarks of the irradiated mucosa. In our model, a pronounced acute crypt degeneration was seen in the fiber-free diet group, whereas crypt degeneration in the fiber-fed animals appeared more subtle and consistent over time. As a result, animals belonging to the fiber-free group had fewer crypts at six weeks post-irradiation than those consuming dietary fiber, albeit the difference was transient and had disappeared by 18 weeks post-irradiation. Although we cannot completely rule out the possibility that crypt degeneration peaked earlier than one week for the fiber-consuming group, we have previously shown that crypt degeneration is not immediate after four fractions of 8 Gy. We found very few degenerating crypts 24 h after irradiation in animals consuming a standard chow diet containing 13 percent dietary fiber [24]. A considerable amount of crypt degeneration was instead found at one week post-irradiation [24], similar to the peak seen in the animals fed the no-fiber diet. In the standard chow, the content (fraction) of dietary fiber was approximately the same as in the bioprocessed oat bran diet (13% vs. 15%) but consisted of nearly 85% insoluble fiber with low levels of β-glucans (0.6% vs. 28%). High levels of easily fermentable fiber might thus be required for reducing the pace of crypt degeneration and “flattening the curve” after irradiation. Nagai et al. used a ^60^Co-γ-irradiator to deliver gamma-radiation to the abdomen of rats fed dietary fiber from sugar beet, or no fiber, and quantified the number of aberrant crypts at 5, 9 and 15 weeks. The main fiber types in sugar beet are insoluble hemi-cellulose and soluble pectin, with two-thirds of the fiber content being insoluble. They found a reduction of aberrant crypts at 5 and 9 weeks compared to animals fed a fiber-free diet [25]. The discrepancy concerning the observations of crypt loss at later versus earlier timepoints between the Nagai et al. study and the current study could be due to many effect-modifying factors, such as the amount and type of fiber (sugar beet converted into high amounts of acetic acid versus oats converted into high amounts of butyric acid), the higher dose and the more intense fractionation schedule in the current study (4 Gy × 3 versus 8 Gy × 4), as well as the species used (rat versus mouse). Another study performed by Sureban et al. concluded that soluble pectin reduced acute crypt loss in mice subjected to 14 Gy of total body irradiation [9]. Both studies also linked the immune system to the protective effects of dietary fiber. Taken together with our own findings, there is thus strong evidence that dietary fiber is able to reduce irradiation-induced crypt loss, but probably only transiently. We believe that the initial DNA damage caused by ionizing irradiation will eventually deplete the crypt stem cells and cause the crypt to collapse, even if the process may be slowed by a high consumption of fermentable dietary fiber. Nevertheless, “flattening the curve” with regard to crypt degeneration could still be important, since it could extend the therapeutic window after pelvic radiotherapy. 

One should consider that the colon is growing even in adult animals, and fewer crypts in a treatment group does not necessarily reflect crypt loss but may also reflect a reduced growth rate. In the sham-irradiated animals, the bioprocessed oat bran increased the number of crypts over time compared to the fiber-deprived animals. The number of crypts in sham-irradiated animals on a fiber-rich diet increased by 44% between week 1 and week 18, while the same increase was only 16% for the fiber-deprived animals. The aforementioned study by Sureban et al. in 2015 showed that one of the intestine’s natural ways of growth, crypt cell proliferation, was stimulated by dietary fiber consumption after irradiation [9]. We found that, although irradiation triggered proliferation in both dietary groups at one week post-irradiation, only the group consuming bioprocessed oat bran had sustained proliferative activity at six weeks post-irradiation. Notably, in an earlier study we did not find an increase in cell proliferation after irradiation at the same time points in mice on regular standard chow [24], and, in contrast to other reports, neither the presence nor the absence of dietary fiber in the current study altered the proliferation rates in sham-irradiated mice at any time-point. The composition of the microbiota plays a role in the proliferative capacity of the crypt after injury and may underlie these findings [26,27]. A recent study in mice showed that irradiation causes a negative shift in bacterial composition in the colon six weeks after irradiation [28]. Results from our own laboratory indicated that the serum cytokine profiles of fiber-consuming and fiber-free animals were similar at one week post-irradiation but clearly separated at six weeks, with more pro-inflammatory cytokines at six weeks [16]. A shift in microbiota composition and the expression of pro-inflammatory cytokines at six weeks could have reduced the capacity of fiber-depleted animals to respond to injury with increased cell proliferation, although such a connection remains to be demonstrated.

A mechanism of growth and repair that could be responsible for the increase in crypt numbers besides crypt proliferation is crypt fission, a process where an entire crypt will divide into two or more new crypts. We are only aware of one previous publication in addition to our own [24] in which crypt fission has been described as a repair mechanism after irradiation [29]. Crypt fission can occur independently from cell proliferation [30], and we have found crypt fission to outlast crypt proliferation in response to irradiation [24]. Here, we quantified the number of crypt fissions per circumference at the various time points and found higher levels of crypt fission in mice on a fiber-rich diet when compared to those fed a fiber-free diet at one week and 18 weeks post-irradiation. However, this was only seen in a subset of animals and did not result in any pronounced difference between the two groups. At the 6-weeks timepoint, there was a notably higher baseline in both control groups compared to the other time points. In contrast to the quantifications of cells stained with immunohistochemistry, where parameters such as tissue fixation and antibody lot quality can result in considerable differences in the outcome, the histological quantification of crypt fissions is not very sensitive to alterations in methodological parameters. Thus, the increased crypt fission baseline at the 6-week time point compared to the other time points is unlikely to be a result of processing differences between the time points. Instead, it could be a time-dependent response to a profound change in diet (transferring from normal chow to the experimental diets), where both diets had a stimulating effect. As previously mentioned, very little is known about crypt fission, and, although reports exist in which the investigators noted a stimulation of crypt fission by dietary fiber [12], the underlying mechanisms are essentially unknown. Another aspect to consider is the recent discovery of “crypt fusion” (the process of two crypts merging into one single crypt) [31,32]. Crypt fusion is difficult to discern from crypt fission and can occur simultaneously after injury as a mechanism to remove, or rescue, dysfunctional crypts by merging. In this study, we cannot exclude that a portion of the crypt fissions were actually fusions and thus resulted in the removal, not the addition, of crypts. This could explain why there was no correlation between crypt fissions and the number of crypts. Nevertheless, both crypt fission and fusion are, in contrast to cell proliferation, largely unexplored as a means of stimulating repair after injury, and it could prove very fruitful to explore their potential concerning mucosal repair after pelvic radiotherapy.

We observed an increase in the number of mucosal macrophages in irradiated animals compared to sham-irradiated animals, regardless of diet. Macrophages are not believed to propagate in the colonic wall, but they are instead continuously recruited from the blood stream [33]. We have previously shown that there is macrophage infiltration into the colorectal mucosa of irradiated mice on a standard chow diet. This infiltration was found to occur around six weeks after irradiation and was persistent for many months [24]. That the number of macrophages appeared unaffected by the various peaks of crypt degeneration and did not correlate with bacterial presence suggests that they were present for another purpose than scavenging debris or patrolling for intruders. In studies on the small intestine, macrophage WNT signaling was found to promote mucosal repair after irradiation [34]. It is thus possible that the chronic presence of macrophages in the colonic mucosa of our mice reflects a regenerative activity. Just as with crypt fission, this finding warrants further exploration, as it could have a significant therapeutic value. 

Despite the accumulated knowledge from over 100 years of treating cancers with radiation and the countless technical improvements since the beginning of radiation oncology, radiation-induced injury to the surrounding non-tumorigenic tissue is still a treatment-limiting issue. Preventing normal-tissue injury to the intestine after pelvic radiotherapy is imperative if we are to curb the growing number of pelvic cancer survivors that live with a chronic disability as a result of their cancer treatment. Nutritional studies in humans, such as the one conducted in pelvic cancer survivors by Wedlake et al. that produced some encouraging results regarding dietary fiber intake [6], are notoriously difficult to execute due to factors such as adherence and correct estimation of intake. In addition, there is a limited possibility to collect samples. In that aspect, nutritional studies in animal models, although far from perfect, have some clear advantages. Many of the effect-modifiers and confounders can be controlled, and samples more readily taken. Although dietary fiber intake in this study could not prevent crypt loss, a typical pathological hallmark of the irradiated mucosa, our findings provide ample evidence that dietary modifications should be taken into consideration when developing strategies for protecting and restoring bowel health during and after pelvic radiotherapy. We found that dietary fiber possessed the capacity to modify the onset, timing and intensity of several intestinal pathophysiological processes and repair mechanisms after radiation and prevented a late, perhaps chronic, bacterial invasion of the mucosa. We also profiled the proinflammatory signature in serum after irradiation and showed that it was determined by the diet. This does not mean that we now know what dietary advice to give to pelvic cancer patients. Foods rich in dietary fiber, including the bioprocessed oat bran used here, contain many bioactive compounds that also could be of importance. There is a lot to learn about the impact of specific dietary fibers and other substances, their composition in various dietary items and their interaction with key players such as the microbiota, the immune system and crypt stem cells. Nevertheless, our findings suggest that it may be possible, by fairly simple and inexpensive strategies, to interfere with the pathophysiological process that develops in the mucosa after pelvic radiotherapy. With further knowledge growth concerning the intake of various food items and their impact on bowel health, we may get means other than dose reduction to protect intestinal health in pelvic cancer survivors.

## 4. Materials and Methods

### 4.1. Animals

All experimental animal procedures were approved by the Gothenburg Committee of the Swedish Animal Welfare Agency (application number 1458-2018) and performed in full compliance with Swedish animal protection legislation. The experiments were performed on male C57BL/6J mice from Charles River Laboratories (Sulzfeld, Germany), with the dietary interventions starting when the mice were 9 weeks old. The mice were housed five to a cage under the following standard conditions: constant temperature (20 °C) with 42% relative humidity and a 12-h day/light cycle. Mice had free access to the assigned diet and water throughout the experiment. 

### 4.2. Experimental Design

An overview of the experimental design is shown in Figure 1A. To determine the dietary effect on mouse colorectum after irradiation, mice on standard chow were switched to diets containing either a high percentage of dietary fiber (15% of bioprocessed oat bran; “High-oat” diet) or a fiber-free diet (0% bioprocessed oat bran or other dietary fiber; “No-fiber” diet) two weeks before irradiation. For each timepoint, eight cages housing five mice each were randomly assigned to 4 groups (*n* = 10 in each diet group): (1) High-oat diet + irradiation, (2) High-oat diet + sham-irradiated, (3) No-fiber diet + irradiation and (4) No-fiber diet + sham-irradiated. Two weeks into the dietary experiment, the mice were irradiated with four fractions of 8 Gray (Gy) and observed once a week for changes in weight, appearance and for any development of symptoms such as anal redness and excessive mucus discharge. The mice were euthanized at 1, 6 and 18 weeks from the last fraction of radiation.

### 4.3. Diets

We opted for a diet with a 15% fiber content, which is higher than the recommended daily intake for humans in Scandinavia (25–35 g of fiber per day, corresponding to approximately 8% of the total daily food intake; Nordic Nutrition Recommendations, NNRs 2012) but within a relevant range. Bioprocessed oat bran was provided by Glucanova AB (Lund, Sweden) and prepared according to European patent # 2996492. The characterization of the product can be seen in Table 1A. The peak molecular weight of the β-glucan fraction was 101,000 ± 11,000 Daltons. The basal mixture (TD.160816) to which the bioprocessed oat bran and/or starch was added was custom made by Envigo Teklad Diets (Madison, WI, USA) in powdered form. The purified cornstarch (Cargill’s C*Gel 03401) was provided by Caldic Ingredients Sweden AB (Malmö, Sweden). The added starch ensured that both diets were similar with regard to caloric value. Both diets were provided in a porridge-like consistency. The composition of experimental diets is listed in Table 1B,C. 

### 4.4. Irradiation Procedure

Our method of small-field irradiation restricted to the colorectal area has been described in detail previously [15]. Briefly, at 11 weeks of age, mice were anesthetized with isoflurane and placed in a silicon mold to ensure identical positioning between subjects and fractions. The 3 × 3 cm^2^ radiation field was placed so that approximately 1.5 cm of the distal colon was irradiated (Figure 1A). Care was taken to exclude the spinal cord, testicles and other body parts of the mice from the radiation field. Irradiation was delivered using a linear accelerator (Varian TrueBeam; Varian Medical Systems Inc., Charlottesville, VA, USA) with 6 MV nominal photon energy. A total of 32 Gy in 4 fractions of 8 Gy, with a dose rate of 5.9 Gy/min, was delivered at 12-h intervals. Sham-irradiated mice were placed under the linear accelerator but received anesthesia only. The rationale for the chosen intervals, fractions and dose was as follows: in the clinic, radiation is usually delivered at 24-h intervals. In mice, DNA repair has been shown to be completed after 5 h [35], and mouse crypt stem cells cycle several times faster than human crypt stem cells [36]. Thus, shortening the intervals to 12 h did not impact the relevance of the model negatively and allowed us to give a higher number of fractions in a shorter period of time, which was necessary due to the limited access to the clinic’s linear accelerators (e.g., weekends). We have previously studied the outcome of different radiation schedules and found 8 × 4 Gy to produce a mucosal pathophysiology similar to that of irradiated human cancer survivors while preserving the animal’s general health and life span [24,37]. 

### 4.5. Collection and Processing of Tissue

The mice were anesthetized with isoflurane (Isoba^®^ vet, MSD Animal Health, Buckinghamshire, UK); the abdomen opened and the distal 7 mm of the irradiated colorectal region (from the anus and proximally) was excised and fixed in methacarn (methanol-Carnoy) for 24 h at room temperature. The shape of the tissue was preserved by inserting a thin, soft silicone tube (OD 1.19 mm ID 0.64 mm, AgnThos, Lidingö, Sweden) rectally. The silicone tubing was carefully removed after fixation and before dehydration and paraffin embedding. All sections were cut in a similar manner using a Leica sliding microtome (Leica RM2235, Leica Biosystems, Wetzlar, Germany). The sections were cut at 4 μm and mounted on slides in a 1:6 series, ensuring that each section mounted on a slide contained only one layer of cells and was well separated in the tissue from the preceding section.

### 4.6. Immunohistochemistry and Histochemistry

For immunohistochemistry, the sections were dewaxed in xylene, rehydrated in series of alcohols and boiled in citrate buffer (pH 6) in a pressure cooker for 3 min for antigen retrieval, followed by 0.6% H_2_O_2_ to inactivate endogenous peroxidase. The sections were then blocked with blocking buffer (TBS containing 0.1% TritonX-100 and 3% donkey serum) for one hour in RT. After incubation with the primary antibodies anti-rabbit Ki-67 (1:150, Merck Millipore, Burlington, MA, USA) or anti-rabbit Iba-1 (1:2000, Wako Chemicals, Neuss, Germany) overnight at +4 °C, the slides were incubated in peroxidase donkey-anti-rabbit secondary antibody (1:250; Jackson ImmunoResearch, Cambridgeshire, UK). After incubation, the antigen was visualized by developing in DAB (Saveen Werner AB, Malmö, Sweden). The sections were then dehydrated in an alcohol series, cleared in xylene and coverslipped in X-TRA-kitt mounting medium (Medite GmbH, Burgdorf, Germany). Verhoeff’s elastic stain was used for the morphometric analyses using tracing or visual crypt and crypt fission counts since it, besides staining nuclei and elastic fibers dark, exposes the gross morphology well by separating collagen (red) from muscle fibers (yellow). It also worked well with the fixation method used. The sections were dewaxed, rehydrated and immersed in Verhoeff’s solution, followed by a rinse in tap and distilled water. After rinsing in distilled water, the sections were differentiated in a 2% ferric chloride solution followed by a 5% sodium thiosulfate solution and rinsed in tap water and distilled water before counterstaining with Van Gieson. After counterstaining, the sections were dehydrated in an alcohol series and clearing in Xylene before mounting and coverslipping with Pertex (Histolab AB, Askim, Sweden).

### 4.7. Fluorescence-In Situ Hybridization to Detect Bacteria

The protocol is described in detail in [38]. The slides were heated at 60 °C for 10 min, rehydrated and dewaxed in EtOH series and xylene and then allowed to air-dry. A total of 500 ng of an Alexa 546-conjugated probe (16S rRNA: 5′-GCTGCCTCCCGTAGGAGT-3′, Eurofins Genomics, Ebersberg, Germany) were added to 100 μL pre-warmed hybridization solution (20 mM Tris-HCl pH 7.4, 0.9 M NaCl and 0.1% SDS). In total, 100 μL probe-hybridization solution were added to each slide and covered with a cover glass. The slides were incubated at 50 °C in a humidity chamber overnight. After the hybridization step, the slides were rinsed for 10 min at 50 °C in pre-warmed wash buffer (20 mM Tris-HCl pH 7.4, 0.9 M NaCl) before being rinsed with H_2_O and PBS for 1 min each. The slides were coverslipped in ProLong™ Gold Antifade Mountant with DAPI (Molecular Probes, Eugene, OR, USA).

### 4.8. Serum Cytokine Analysis

The serum cytokine levels for 23 cytokines were measured using the Bio-Plex mouse cytokine 23-plex Assay (Bio-Rad Laboratories AB, Solna, Sweden). The cytokines were: (IL)-1α, IL-1β, IL-2, IL-3, IL-4, IL-5, IL-6, IL-9, IL-10, IL-12p40, IL-12p70, IL-13, IL-17A, eotaxin, granulocyte colony-stimulating factor (G-CSF), granulocyte-macrophage colony-stimulating factor (GM-CSF), interferon (IFN)-γ, keratinocyte-derived chemokine (KC), monocyte chemoattractant protein-1 (MCP-1), macrophage inflammatory protein (MIP)-1 α, MIP-1 β, regulated on activation normal T-cell expressed and secreted (RANTES) and tumor necrosis factor (TNF)-α. The detailed method for measuring serum cytokines and the acquired data have been published previously [16].

### 4.9. Assessment of Crypt Fission and Crypt Degeneration

For the assessment of crypt fission and crypt degeneration, sections stained with Verhoeff Elastic stain were used. A total of 6 sections approximately 24 μm apart from each other were analyzed per animal in 40× magnification using a Leica DM6000B microscope (Leica Microsystems, Wetzlar, Germany). A crypt that was dividing at the bottom, forming two or more new daughter crypts but with a common opening towards the lumen, was counted as one crypt fission. A crypt that had a closed opening to the lumen, was dilated and only had a thin cell wall was considered a degenerating crypt. 

### 4.10. Crypt Number

To assess the dietary effect on colon crypt survival after pelvic radiation, the number of crypts per circumference was quantified. All of the crypts in three circumferences spaced approximately 48 µm apart were counted per animal, and the average number of crypts per animal was calculated and reported. 

### 4.11. Quantification of Proliferating Cells

The proliferation marker Ki-67 was used to quantify the proliferating cells in the crypts. Only crypts that were cut perpendicularly along their entire axis and had a clear opening towards lumen were included in the counting. A total of 24 crypts from two circumferences spaced approximately 72 µm apart were counted. The average number of Ki67^+^ cells per crypt was calculated and reported.

### 4.12. Quantification of Colonic Macrophages

Macrophages were identified using an antibody against the ionized calcium-binding adapter molecule 1 Iba1. Iba1 is a pan-macrophage marker strongly expressed in colonic macrophages, and, while it can also be expressed in a subset of dendritic cells, the majority of the Iba1^+^ cells in our tissues could be expected to be macrophages [39,40]. Cells positive for Iba1 were quantified using a modified stereology-based method and a Leica DM6000B microscope equipped with a semi-automated stage and the Stereo Investigator software (MBF Bioscience, Williston, VT, USA). All of the slides were blinded and coded before the count. Three circumferences separated by approximately 48 µm were analyzed per animal. The mucosal area was traced at 5× magnification, and Iba1-positive cells were quantified at 40× magnification as follows: a 150 × 150 µm grid was randomly placed over the traced area with a counting of 50 × 50 µm placed at each intersection. Approximately 50 counting frames per section were analyzed for the number of Iba1 cells according to set counting rules [41]. Based on the number of Iba-1 cells counted, the software estimated the total number of Iba-1 cells present in the traced area. The average number from three circumferences was reported.

### 4.13. Quantification of Bacteria

Bacteria were visible in the tissue as strongly labeled rod- or sphere-shaped bodies. Using 63× magnification, all strongly labeled bacteria in the mucosa and submucosa were counted in three circumferences separated by approximately 48 µm. The average number of bacteria per section was calculated by dividing the total number of bacteria by the number of sections counted.

### 4.14. Statistical Analysis

Statistical tests for the bar graphs (Figure 1, Figure 2, Figure 3, Figure 4, Figure 5 and Figure 7) were performed using the GraphPad Prism 8 software (GraphPad Software LLC, San Diego, CA, USA), and data were expressed as mean + S.E.M. A *p*-value equal to or below 0.05 was considered statistically significant. Without enough information to perform a power analysis, sample size justification (*n* = 10 per treatment and time point) was made based on our previous experience using the mouse model. If the tissue was in poor condition, it was excluded, giving a final *n* of 6–10. Descriptive statistics are presented in Table 2. If a data set passed the Shapiro-Wilks test for normal distribution, either as non-transformed or as log-transformed, a two-way analysis of variance (ANOVA) was applied, with diet and irradiation as factors, followed by a Bonferroni post-hoc test. If a data set did not pass the Shapiro-Wilks test for normal distribution, a Kruskal-Wallis test on ranks followed by Dunn’s post-hoc was performed for all three time points for that specific parameter. We did not perform comparisons between the time points to avoid the introduction of unknown confounding factors that may arise when experiments are executed separately, such as mouse batch differences and variations in the quality of reagents. For example, despite the careful documentation and execution of each experimental step to ensure reproducibility, the 6-weeks timepoint displayed a lower base line concerning the number of Ki67^+^ cells than the other two timepoints (Figure 4). For the growth curves of the 18-weeks mice in Figure 1B, three groups (diet and irradiation as between-subjects factors and time as within-subjects factor) were compared using a three-way mixed ANOVA with a Geisser-Greenhouse correction for the lack of sphericity. Statistical analyses are presented in Table 3.

The statistical analysis for the cytokine expression data presented in Figure 6 was conducted as follows: in order to determine a possible association between treatment (irradiation or sham-irradiation and diet) and the serum expression levels of 23 cytokines measured previously [16] at 18 weeks post-irradiation (*n* = 6 per treatment group), the mice were clustered with respect only to cytokine levels, and the association between cluster affiliation and treatment was subsequently investigated. Prior to calculating the distance matrix to be used in the cluster analysis, the cytokine measurements were normalized cytokine-wise in order to avoid any single cytokine making a disproportionate contribution. Based on this normalized data, a distance matrix with respect to mice was calculated using the Euclidean distance. The calculation of the distance matrix was performed using the R function dist(), the remaining missing values being handled by the internal rescaling method of that function. A hierarchical cluster structure was then assessed using complete linkage via the R function hclust(). Inspection of the cluster tree structure suggested extracting three clusters. Finally, the association between the clusters produced and the treatment and diet categories was examined using Fisher’s exact test.

## Figures and Tables

**Table 1:** Diets. (A) Bioprocessed oat bran specifications. (B) Diet compositions. (C) Ingredients basal diet mixture. * Dry-weight basis. # Tertiary butylhydroquinone.

**(A)**		
**Nutritional information per 100 g of powder**		
**Energy (average)**		**280 kcal**
Moisture		8–9 g
Protein		23 g
Carbohydrate total -of which sugars		9 g 6 g
Fat total		5 g
Dietary Fiber total -of which β-glucan		52 g 28 g
**(B)**		
**Diet composition (%)**	**High oat** * 15% fiber	**No fiber** *0% fiber
Bioprocessed oat bran	28.8 *	0
Corn starch	4.7	33.5
Basal diet mixture	66.5	66.5
**Total**	**100**	**100**
**(C)**		
**Basal diet mixture (g/100 g High oat or No fiber diet)**		
Casein		13.3
DL-Methionine		0.2
Corn starch		25.0
Maltodextrin		8.7
Sucrose		10.6
Olive oil		4.7
Vitamin mixture		1.0
Choline bitartrate		0.2
TBHQ ^#^		0.001
Mineral mixture		1.3
Calcium phosphate		1.1
Calcium carbonate		0.4
**Total weight (g)**		**66.5**

**Table 2:** Descriptive statistics.

**1 week** **Post-Irradiation**		**High-oat**						**No-fiber**					
		**Sham-irr**			**Irr**			**Sham-irr**			**Irr**		
**Parameter**	**Figure**	**Mean**	**± SEM**	**N**	**Mean**	**± SEM**	**N**	**Mean**	**± SEM**	**N**	**Mean**	**± SEM**	**N**
Degenerating crypts	2A	0	0	10	6.2	1.6	9	0	0	10	24.6	4.8	7
Crypt fisson	3A	0.1	0.1	10	5	1.4	10	0	0	10	2.1	0.5	10
ki67^+^ cells per crypt	4A	9.6	0.8	9	20.8	2.1	7	9.2	0.7	10	19.9	2.4	9
Crypts/circumference	5A	140.1	3.3	8	74	3.8	8	129.7	4.4	8	64.7	5.2	10
Bacteria/circumference	6A	94	8.8	10	91	12	10	91	4.8	10	92	6.1	10
Iba1^+^ cells/circumference	7A	579.9	79.5	10	1378	250.7	8	999.6	256.5	10	1271	215.4	9
**6 weeks** **Post-irradiation**		**High-oat**						**No-fiber**					
		**Sham-irr**			**Irr**			**Sham-irr**			**Irr**		
**Parameter**	**Figure**	**Mean**	**± SEM**	**N**	**Mean**	**± SEM**	**N**	**Mean**	**± SEM**	**N**	**Mean**	**± SEM**	**N**
Degenerating crypts	2B	0.0	0.0	10	8.3	2.7	9	0.0	0.0	10	3.5	1.2	10
Crypt fisson	3B	1.7	0.5	10	3.9	1.1	9	4.1	0.8	10	5.6	1.2	9
ki67^+^ cells per crypt	4B	3.7	0.2	6	6.4	0.8	9	3.3	0.2	7	3.6	0.4	9
Crypts/circumference	5B	166.2	7.4	6	144.7	5.7	8	144.5	6.3	10	101.0	9.5	9
Iba1^+^ cells/circumference	7B	239.2	20.7	10	318.8	34.3	9	201.2	19.7	10	356.5	36.9	7
**18 weeks** **Post-irradiation**		**High-oat**						**No-fiber**					
		**Sham-irr**			**Irr**			**Sham-irr**			**Irr**		
**Parameter**	**Figure**	**Mean**	**± SEM**	**N**	**Mean**	**± SEM**	**N**	**Mean**	**± SEM**	**N**	**Mean**	**± SEM**	**N**
Bodyweight (grams)	1C	34.2	0.5	10	32.5	0.5	10	41.1	1.9	10	41.8	1.1	10
Degenerating crypts	2C	0.0	0.0	10	1.2	0.5	10	0.0	0.0	10	1.3	0.7	10
Crypt fisson	3C	0.7	0.3	10	2.8	0.8	10	0.0	0.0	10	1.7	0.5	10
ki67^+^ cells per crypt	4C	8.6	1.0	10	10.3	1.3	10	9.9	1.2	10	12.9	1.2	10
Crypts/circumference	5C	202.6	7.8	9	107.6	4.7	7	158.5	10.3	10	97.6	4.3	10
Bacteria/circumference	6B	17	2.5	10	19	1.8	8	25	0.94	10	159	18	9
Iba1^+^ cells/circumference	7C	232.5	21.3	10	294.8	33.6	8	209.7	25.7	10	280.2	39.5	10

**Table 3:** Statistical analysis.

**1 week post-irradiation**					**Dunn’s post-hoc**		**Dunn’s post-hoc**	
					**H-oat irr vs. sham-irr**	**No fiber irr vs. sham-irr**	**H-oat irr vs.** **No fiber-irr**	**H-oat sham-irr vs. No fiber sham-irr**
**Parameter**	**Figure**	***p* value, Kruskal-Wallis test**			*p*-value	*p*-value	*p*-value	*p*-value
Degenerating crypts	2A	<0.0001 ****			0.0191 *	<0.0001 ****	0.2644	>0.9999
Crypt fisson	3A	<0.0001 ****			0.001 ***	0.0034 **	>0.9999	>0.9999
Crypts/circumference	5A	<0.0001 ****			0.0025 **	0.0017 **	>0.9999	>0.9999
Bacteria/circumference	6A	0.9953			N/A	N/A	N/A	N/A
		***p*-value, Two-way ANOVA**			**Bonferroni’s post-hoc**		**Bonferroni’s post-hoc**	
**Parameter**	**Figure**	Irradiation	Diet	Interaction	*p*-value	*p*-value	*p*-value	*p*-value
Ki67^+^ cells per crypt	4A	<0.0001 ****	0.6883	0.8585	0.0002 ***	0.0001 ****	>0.9999	>0.9999
Iba1^+^cells/circumference	7A	0.6054	0.1290	0.5243	N/A	N/A	N/A	N/A
**6 weeks post-irradiation**					**Dunn’s post-hoc**		**Dunn’s post-hoc**	
					**H-oat irr vs. sham-irr**	**No fiber irr vs. sham-irr**	**H-oat irr vs.** **No fiber-irr**	**H-oat sham-irr vs. No fiber sham-irr**
**Parameter**	**Figure**	***p* value, Kruskal-Wallis test**			*p*-value	*p*-value	*p*-value	*p*-value
Degenerating crypts	2B	<0.0001 ****			0.0008 ***	0.0205 *	>0.9999	>0.9999
Crypt fisson	3B	0.0545			N/A	N/A	N/A	N/A
Crypts/circumference	5B	0.0022 **			0.5057	0.1155	0.0771	0.2537
		***p*-value, Two-way ANOVA**			**Bonferroni’s post-hoc**		**Bonferroni’s post-hoc**	
**Parameter**	**Figure**	Irradiation	Diet	Interaction	*p*-value	*p*-value	*p*-value	*p*-value
Ki67^+^ cells per crypt	4B	0.0117 *	0.0049 **	0.0378 *	0.0088 **	>0.9999	0.0015 **	>0.9999
Iba1^+^ cells/circumference	7B	0.0002 ***	0.9951	0.1800	0.1700	0.0022 **	>0.9999	>0.9999
**18 weeks post-irradiation**					**Dunn’s post-hoc**		**Dunn’s post-hoc**	
					**H-oat irr vs. sham-irr**	**No fiber irr vs. sham-irr**	**H-oat irr vs.** **No fiber-irr**	**H-oat sham-irr vs. No fiber sham-irr**
**Parameter**	**Figure**	***p* value, Kruskal-Wallis test**			*p*-value	*p*-value	*p*-value	*p*-value
Degenerating crypts	2C	0.0408 *			0.1209	0.2414	>0.9999	>0.9999
Crypt fisson	3C	0.0011 **			0.1212	0.0157 *	>0.9999	0.4556
Crypts/circumference	5C	<0.0001 ****			0.0008 ***	0.0025 **	>0.9999	0.6512
Bacteria/circumference	6B	<0.0001 ****			>0.9999	0.0451 *	0.0005 ***	0.1234
		***p*-value, Two-way ANOVA**			**Bonferroni’s post-hoc**		**Bonferroni’s post-hoc**	
**Parameter**	**Figure**	Irradiation	Diet	Interaction	*p*-value	*p*-value	*p*-value	*p*-value
Body weight (grams)	1C	0.6889	<0.0001 ****	0.3026	>0.9999	>0.9999	<0.0001 ****	0.0006 ***
ki67^+^ cells per crypt	4C	0.0641	0.1077	0.5966	N/A	N/A	N/A	N/A
Iba1^+^ cells/circumference	7C	0.0386 *	0.5489	0.8944	0.6982	0.4198	>0.9999	>0.9999

* *p* < 0.05; ** *p* < 0.01; *** *p* < 0.001; **** *p* < 0.0001.

## Data Availability

The data that support the findings of this study are available from the corresponding author upon reasonable request.

## References

[1] Andreyev H.J., Wotherspoon A., Denham J.W., Hauer-Jensen M. (2011). “Pelvic radiation disease”: New understanding and new solutions for a new disease in the era of cancer survivorship. Scand. J. Gastroenterol..

[2] Steineck G., Skokic V., Sjöberg F., Bull C., Alevronta E., Dunberger G., Bergmark K., Wilderäng U., Oh J.H., Deasy J.O. (2017). Identifying radiation-induced survivorship syndromes affecting bowel health in a cohort of gynecological cancer survivors. PLoS ONE.

[3] Hur W., Yoon S.K. (2017). Molecular Pathogenesis of Radiation-Induced Cell Toxicity in Stem Cells. Int. J. Mol. Sci..

[4] Potten C.S., Grant H.K. (1998). The relationship between ionizing radiation-induced apoptosis and stem cells in the small and large intestine. Br. J. Cancer.

[5] Francois A., Milliat F., Guipaud O., Benderitter M. (2013). Inflammation and immunity in radiation damage to the gut mucosa. BioMed Res. Int..

[6] Wedlake L., Shaw C., McNair H., Lalji A., Mohammed K., Klopper T., Allan L., Tait D., Hawkins M., Somaiah N. (2017). Randomized controlled trial of dietary fiber for the prevention of radiation-induced gastrointestinal toxicity during pelvic radiotherapy. Am. J. Clin. Nutr..

[7] Brotherton C.S., Taylor A.G., Bourguignon C., Anderson J.G. (2014). A high-fiber diet may improve bowel function and health-related quality of life in patients with Crohn disease. Gastroenterol. Nurs..

[8] Nyman M., Nguyen T.D., Wikman O., Hjortswang H., Hallert C. (2020). Oat Bran Increased Fecal Butyrate and Prevented Gastrointestinal Symptoms in Patients with Quiescent Ulcerative Colitis—Randomized Controlled Trial. Crohn’s Colitis 360.

[9] Sureban S.M., May R., Qu D., Chandrakesan P., Weygant N., Ali N., Lightfoot S.A., Ding K., Umar S., Schlosser M.J. (2015). Dietary Pectin Increases Intestinal Crypt Stem Cell Survival following Radiation Injury. PLoS ONE.

[10] Desai M.S., Seekatz A.M., Koropatkin N.M., Kamada N., Hickey C.A., Wolter M., Pudlo N.A., Kitamoto S., Terrapon N., Muller A. (2016). A Dietary Fiber-Deprived Gut Microbiota Degrades the Colonic Mucus Barrier and Enhances Pathogen Susceptibility. Cell.

[11] Bach Knudsen K.E., Laerke H.N., Hedemann M.S., Nielsen T.S., Ingerslev A.K., Gundelund Nielsen D.S., Theil P.K., Purup S., Hald S., Schioldan A.G. (2018). Impact of Diet-Modulated Butyrate Production on Intestinal Barrier Function and Inflammation. Nutrients.

[12] McCullogh J.S., Ratcliffe B., Mandir N., Carr K.E., Goodlad R.A. (1998). Dietary fibre and intestinal microflora: Effects on intestinal morphometry and crypt branching. Gut.

[13] Sengupta S., Tang C.L., Wong C.S., Tjandra J.J., Gibson P.R. (2002). Colonic epithelial atrophy induced by a fibre-free diet in rats is reversed by minimal amounts of luminal butyrate, but only in the short term. ANZ J. Surg..

[14] Ahlin R., Sjoberg F., Bull C., Steineck G., Hedelin M. (2018). [Differing dietary advice are given to gynaecological and prostate cancer patients receiving radiotherapy in Sweden]. Lakartidningen.

[15] Bull C., Malipatlolla D., Kalm M., Sjoberg F., Alevronta E., Grander R., Sultanian P., Persson L., Bostrom M., Eriksson Y. (2017). A novel mouse model of radiation-induced cancer survivorship diseases of the gut. Am. J. Physiol. Gastrointest. Liver Physiol..

[16] Patel P., Malipatlolla D.K., Devarakonda S., Bull C., Rascón A., Nyman M., Stringer A., Tremaroli V., Steineck G., Sjöberg F. (2020). Dietary Oat Bran Reduces Systemic Inflammation in Mice Subjected to Pelvic Irradiation. Nutrients.

[17] Jang H., Lee J., Park S., Kim J.S., Shim S., Lee S.B., Han S.H., Myung H., Kim H., Jang W.S. (2019). Baicalein Mitigates Radiation-Induced Enteritis by Improving Endothelial Dysfunction. Front. Pharmacol..

[18] Bensemmane L., Squiban C., Demarquay C., Mathieu N., Benderitter M., Le Guen B., Milliat F., Linard C. (2021). The stromal vascular fraction mitigates radiation-induced gastrointestinal syndrome in mice. Stem Cell Res. Ther..

[19] Schroeder B.O., Birchenough G.M.H., Stahlman M., Arike L., Johansson M.E.V., Hansson G.C., Backhed F. (2018). Bifidobacteria or Fiber Protects against Diet-Induced Microbiota-Mediated Colonic Mucus Deterioration. Cell Host Microbe.

[20] Eftychi C., Schwarzer R., Vlantis K., Wachsmuth L., Basic M., Wagle P., Neurath M.F., Becker C., Bleich A., Pasparakis M. (2019). Temporally Distinct Functions of the Cytokines IL-12 and IL-23 Drive Chronic Colon Inflammation in Response to Intestinal Barrier Impairment. Immunity.

[21] Khanna R., Afif W. (2021). Ustekinumab for Ulcerative Colitis. Gastroenterology.

[22] Nizzoli G., Burrello C., Cribiu F.M., Lovati G., Ercoli G., Botti F., Trombetta E., Porretti L., Todoerti K., Neri A. (2018). Pathogenicity of In Vivo Generated Intestinal Th17 Lymphocytes is IFNgamma Dependent. J. Crohns Colitis.

[23] Chewning J.H., Weaver C.T. (2014). Development and survival of Th17 cells within the intestines: The influence of microbiome- and diet-derived signals. J. Immunol..

[24] Malipatlolla D.K., Patel P., Sjoberg F., Devarakonda S., Kalm M., Angenete E., Lindskog E.B., Grander R., Persson L., Stringer A. (2019). Long-term mucosal injury and repair in a murine model of pelvic radiotherapy. Sci. Rep..

[25] Nagai T., Ishizuka S., Hara H., Aoyama Y. (2000). Dietary sugar beet fiber prevents the increase in aberrant crypt foci induced by gamma-irradiation in the colorectum of rats treated with an immunosuppressant. J. Nutr..

[26] Nowacki M.R. (1993). Cell proliferation in colonic crypts of germ-free and conventional mice—Preliminary report. Folia Histochem. Cytobiol..

[27] Alam M., Midtvedt T., Uribe A. (1994). Differential cell kinetics in the ileum and colon of germfree rats. Scand. J. Gastroenterol..

[28] Gerassy-Vainberg S., Blatt A., Danin-Poleg Y., Gershovich K., Sabo E., Nevelsky A., Daniel S., Dahan A., Ziv O., Dheer R. (2018). Radiation induces proinflammatory dysbiosis: Transmission of inflammatory susceptibility by host cytokine induction. Gut.

[29] Cairnie A.B., Millen B.H. (1975). Fission of crypts in the small intestine of the irradiated mouse. Cell Tissue Kinet..

[30] Berlanga-Acosta J., Playford R.J., Mandir N., Goodlad R.A. (2001). Gastrointestinal cell proliferation and crypt fission are separate but complementary means of increasing tissue mass following infusion of epidermal growth factor in rats. Gut.

[31] Bruens L., Ellenbroek S.I.J., van Rheenen J., Snippert H.J. (2017). In Vivo Imaging Reveals Existence of Crypt Fission and Fusion in Adult Mouse Intestine. Gastroenterology.

[32] Baker A.M., Gabbutt C., Williams M.J., Cereser B., Jawad N., Rodriguez-Justo M., Jansen M., Barnes C.P., Simons B.D., McDonald S.A. (2019). Crypt fusion as a homeostatic mechanism in the human colon. Gut.

[33] Bain C.C., Bravo-Blas A., Scott C.L., Perdiguero E.G., Geissmann F., Henri S., Malissen B., Osborne L.C., Artis D., Mowat A.M. (2014). Constant replenishment from circulating monocytes maintains the macrophage pool in the intestine of adult mice. Nat. Immunol..

[34] Saha S., Aranda E., Hayakawa Y., Bhanja P., Atay S., Brodin N.P., Li J., Asfaha S., Liu L., Tailor Y. (2016). Macrophage-derived extracellular vesicle-packaged WNTs rescue intestinal stem cells and enhance survival after radiation injury. Nat. Commun..

[35] Cai W.B., Roberts S.A., Potten C.S. (1997). The number of clonogenic cells in crypts in three regions of murine large intestine. Int. J. Radiat. Biol..

[36] Potten C.S. (2004). Radiation, the ideal cytotoxic agent for studying the cell biology of tissues such as the small intestine. Radiat. Res..

[37] Steineck G., Bull C., Kalm M., Sjoberg F., Alevronta E., Malipatlolla D.K., Bergmark K., Jeppsson B., Wilderang U., Bjork-Eriksson T. (2017). Radiation physiology—Evidence for a higher biological effect of 24 Gy in four fractions as compared to three. Acta Oncol..

[38] Johansson M.E., Hansson G.C. (2012). Preservation of mucus in histological sections, immunostaining of mucins in fixed tissue, and localization of bacteria with FISH. Methods Mol. Biol..

[39] Kohler C. (2007). Allograft inflammatory factor-1/Ionized calcium-binding adapter molecule 1 is specifically expressed by most subpopulations of macrophages and spermatids in testis. Cell Tissue Res..

[40] Chen Z.W., Ahren B., Ostenson C.G., Cintra A., Bergman T., Moller C., Fuxe K., Mutt V., Jornvall H., Efendic S. (1997). Identification, isolation, and characterization of daintain (allograft inflammatory factor 1), a macrophage polypeptide with effects on insulin secretion and abundantly present in the pancreas of prediabetic BB rats. Proc. Natl. Acad. Sci. USA.

[41] Mouton P.R., Phoulady H.A., Goldgof D., Hall L.O., Gordon M., Morgan D. (2017). Unbiased estimation of cell number using the automatic optical fractionator. J. Chem. Neuroanat..

